# Does size matter? Outcomes following revision total hip arthroplasty with long or primary stems: a systematic review and meta-analysis

**DOI:** 10.1186/s42836-023-00228-w

**Published:** 2024-01-09

**Authors:** Rajpreet Sahemey, Ali Ridha, Alastair Stephens, Muhamed M. Farhan-Alanie, Jakub Kozdryk, Bryan Riemer, Pedro Foguet

**Affiliations:** Department of Trauma & Orthopaedics, University Hospitals Coventry & Warwickshire, Coventry, CV2 2DX UK

**Keywords:** Arthroplasty, Femur, Hip, Outcome, Revision, Meta-analysis

## Abstract

**Background:**

Femoral reconstruction with long stems is widely accepted as the standard in revision total hip arthroplasty (rTHA). However, long stems can be technically challenging to insert and can compromise bone stock for future revision. This study aimed to identify whether there was a difference in outcomes with using a long versus primary or short femoral stem in revision.

**Methods:**

We performed a systematic review and meta-analysis of all articles comparing long and primary stem length in rTHA for Paprosky 1-3B femoral defects. The primary outcome measure was the reoperation rate after rTHA. Secondary outcomes included infection and dislocation rates, periprosthetic fracture, loosening, mortality, and patient-reported outcome measures (PROMs).

**Results:**

The results of 3,102 rTHAs performed in 2,982 patients were reported from 9 eligible studies in the systematic review, of which 6 were included in the meta-analysis. The mean patient age was 67.4 and the mean follow-up lasted 5 years (range, 1–15 years). There was no significant difference in the reoperation rate (odds ratio 0.78; 95% confidence interval, 0.28–2.17, *P* = 0.63). Similarly, there was no significant difference in dislocation or periprosthetic fracture risk. Harris Hip Score was better with primary stems by a mean difference of 14.4 points (*P* < 0.05). Pooled 5-year stem-related survival was 91.3% ± 3.5% (SD) for primary stems and 89.9% ± 6.7% (SD) for long stems.

**Conclusions:**

A primary stem provided non-inferior outcomes compared with long stems in rTHA with Paprosky type 1-3B femoral defects. Primary stems may yield a more straightforward technique and preserve distal bone stock for future revision particularly in younger patients. In older patients with lower functional demands and who would benefit from a decreased risk of complications, a long cemented stem is recommended.

## Background

Primary total hip arthroplasty (THA) remains one of the most commonly performed and successful operations worldwide. Consequently, the incidence of revision total hip arthroplasty (rTHA) is also increasing, accounting for nearly 15% of all THA performed [1–4]. According to registry data, common aetiologies of rTHA include aseptic loosening (39%), prosthetic joint infection (PJI; 20%), and periprosthetic fracture (20%), with revision of the femoral component alone accounting for the most common type of revision procedure (32%) [5].

Traditionally during revision of the femoral component, surgeons use an implant that bypasses the femoral defect by 2 cortical diameters. However, a more appropriate approach to implant selection may depend on the indication for revision and the Paprosky classification of femoral deficiency and bone stock to achieve prosthesis stability [6, 7]. In situations or cases involving severe meta diaphyseal bone loss such as following a periprosthetic fracture or an extensile femoral osteotomy, longer cemented and uncemented stems are typically used with good long-term results and predictable outcomes [8–10]. These allow for bridging of the proximal defects via distal diaphyseal fixation. Also, the use of modular stems allows components to engage and fill the diaphysis and focal bony defects [11, 12].

However, the use of long revision stems can increase the cost and complexity of the procedure as well as increase the risk of intraoperative femoral fracture, stress shielding and reducing distal bone stock available for future reconstruction [13–15]. Considering these factors, the use of primary or “short” stems in rTHA has become more popular in recent years [16]. This systematic review and meta-analysis aimed to compare the use of long versus primary length femoral stems during rTHA in terms of risk of re-revision and complications in patients with Paprosky femoral defect types I to IIIB.

## Methods

### Data sources and search strategy

The study was registered in the International Prospective Register of Systematic Reviews (PROSPERO; CRD42023383375). A systematic search of the literature was performed on 1 December 2022 by 3 authors (R.S., A.R., A.S.) in accordance with the Preferred Reporting Items for Systematic Reviews and Meta-Analyses (PRISMA) guidelines [17]. PubMed, MEDLINE, EMBASE, Cumulative Index to Nursing and Allied Health Literature (CINAHL), and the Cochrane Central Register of Controlled Trials (CENTRAL) databases were searched for relevant articles. The following keywords were used alone or in combination with brackets and Boolean operators (AND, OR, NOT) to help reduce the chances of errors made in syntax: “revision hip”, “long stem”, “short stem”, “primary stem” and “revision stem”. Bibliographies from articles were further scrutinized to identify eligible studies. The primary outcome was the all-cause reoperation rate after rTHA. Secondary outcomes included deep infection, dislocation, periprosthetic fracture, loosening, mortality, and functional outcomes assessed from patient-reported outcome measures (PROMs).

All studies were collated using Rayyan (Rayyan Systems Inc, MA, USA) for the detection of duplicates and initial screening for eligibility based on title and abstract. Full texts were reviewed following which only those articles fulfilling the inclusion criteria were included in the meta-analysis.

### Eligibility criteria

Randomized controlled trials, single group series and cohort studies involving adult patients who had undergone single-stage or staged rTHA for any indication using a long compared with primary or short-length femoral stem were considered eligible for inclusion. Outcomes of interest such as fracture, PJI, reoperation, stem-related survivorship and validated outcome scores were included. The definition for the length of primary and long stems was used within the original study. Where this was not reported, a long stem was defined by consensus as an implant with a length of more than 170 mm from the stem tip to the centre of rotation as many implant manufacturers provide primary stems of up to 165 mm long. No restrictions were imposed on the choice of standard implants (custom and patient-specific implants excluded), surgical technique or use and type of cement. Studies were excluded if, treatment groups were indiscernible or those in which a patient group received an additional intervention (e.g., plate fixation), and those with Paprosky Type IV femoral defects. Studies were also excluded if the article language was not English and translations were not available. Any disagreements regarding study eligibility were resolved by consensus discussion and where necessary escalated to consultation of a fourth reviewer.

### Data extraction and quality assessment

The primary outcome measure was the reoperation rate after rTHA. Secondary outcomes included deep infection and dislocation rates, periprosthetic fracture, loosening, mortality and patient-reported outcome measures (PROMs). The patient demographics were extracted to provide an overview of the population. Age, sex, Paprosky femoral defect type, type of implant used, use of cement, follow-up length and outcomes were obtained. The data were synthesized in narrative and tabular formats.

### Quality assessment

A standardized data extraction form was used. Methodological quality and risk of bias were assessed using the Newcastle–Ottawa Scale for cohort studies [18] and the RoB-2 Risk of Bias tool for randomized controlled trials [19].

### Statistical analysis

We performed a quantitative meta-analysis using R Statistical Software (v4.2.2; R Core Team 2021, Vienna, Austria). Mantel–Haenszel statistics was used to generate odds ratios (ORs) and their corresponding 95% confidence intervals (CIs). Random effects models were used due to the considerable heterogeneity between studies. The I^2^ and τ^2^ were used to calculate heterogeneity. A *P*-value < 0.05 was considered statistically significant. All plots were generated using R.

## Results

### Study selection

**Fig. 1:**
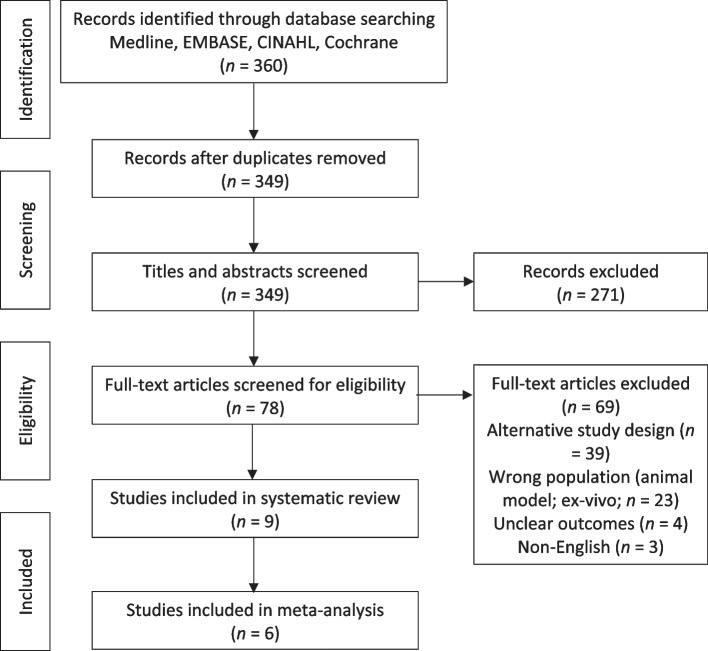
Preferred Reporting Items for Systematic Reviews and Meta-Analyses (PRISMA) flowchart of study selection process

The initial search yielded 360 potentially relevant articles. After removing 11 duplicates, scrutiny of titles and abstracts not meeting the inclusion criteria led to the exclusion of 271 articles. Full-text publications were further assessed and 69 studies were excluded, leaving 9 studies in the systematic review and 6 for meta-analysis. The PRISMA flowchart of the methodology is shown in Fig. 1. The quality of evidence ranged from 1 RCT (randomized controlled trial; level I), 3 prospective cohort studies (level II and III), and 5 retrospective cohort studies (level III and IV), which also included arthroplasty register data. The included studies are detailed in Table 1. The Newcastle–Ottawa Scale used to assess the risk of bias for non-randomised studies is outlined in Table 2. A high risk of bias was detected in the single randomized controlled trial used in this study according to the RoB-2 assessment.

**Table 1:** Summary and main features of the included studies

Author	Year	Study Level, Design	Number of patients/hips	Mean age (years)	rTHA stem size (long/short)	Paprosky femoral defect	Fixation of revised stem	Indication for revision	Mean follow-up (years)	Stem survival (long/short), %
Cameron [20]	2002	II, PC	211/320	68.5	211/109	I-IIIB	320 Cementless	29 GT non-union 18 malposition 16 PPFx 15 other 6 LLD 4 RD 2 stem fracture	7	75/88
Howie et al., [21]	2007	II, PC	211/219	72	137/82	I-IIIB	219 Cementless	163 AL 26 PPFx 7 RD 3 pain 3 stem fracture	6	94/91 *(P* = 0.45)
Li et al., [22]	2016	I, RCT	65/65	65.7	33/32	I-IIIB	65 Cementless	41 AL 12 subsidence 12 PPFx	5	92/86 (*P* > 0.05)
Petrie et al., [23]	2017	II, RC	99/102	67	32/70	I-IIIB	85 cemented 17 cementless	102 PJI	5.5	> 90/ > 90 (*P* > 0.05)
Tetreault et al., [24]	2014	IV, RC	277/277	63	133/144	I-IIIA	277 Cementless	54 PJI 48 AL 8 RD 6 PPFx 2 stem fracture 5 other	4	N/R
Toni et al., [25]	1994	IV, RC	79/79	N/R	6/73	I-IIIB	25 cemented 54 cementless	79 AL	3	N/R
Tsai et al., [26]	2022	III, RC	96/96	61.6	72/24	I-II	24 cemented 72 cementless	96 AL	7	94/92 (*P* = 0.69)
Weiss et al., [27]	2011	IV, RC	1885/1885	74	1073/812	I-IIIB	1073 cemented 812 cementless	1139 AL 438 PPFx 19 PJI 27 RD	3.8	97/95
Willems et al., [28]	2022	II, PC	59/59	65.5	30/29	II	59 cementless	26 AL 21 PJI 6 malposition 3 wear 2 RD	3.5	87/97

*AL* aseptic loosening, *GT* greater trochanter, *LLD* leg length discrepancy, *N/R* not reported, *PC* prospective cohort, *PJI* prosthetic joint infection, *PPFx* peri-prosthetic fracture, *RC* retrospective cohort, *RCT* randomised controlled trial, *RD* recurrent dislocation, *rTHA* revision total hip arthroplasty

**Table 2:** Newcastle–Ottawa Scale for assessing the quality of observational studies

Author	Year	Study	Selection (/4)	Comparability	Exposure	Total stars
Tsai et al., [26]	2022	Retrospective, cohort	★★★★	★	★★★	8
Willems et al., [28]	2022	Prospective, cohort	★★★★	★	★★★	8
Cameron [20]	2002	Prospective, cohort	★★★★		★★★	7
Howie et al., [21]	2007	Prospective, cohort	★★★★		★★★	7
Petrie et al., [23]	2017	Retrospective, cohort	★★★★		★★★	7
Tetreault et al., [24]	2014	Retrospective, cohort	★★★★		★★	6
Toni et al., [25]	1994	Retrospective, cohort	★★★★		★★	6
Weiss et al., [27]	2011	Retrospective, cohort	★★★★		★★	6

### Study characteristics

**Fig. 2:**
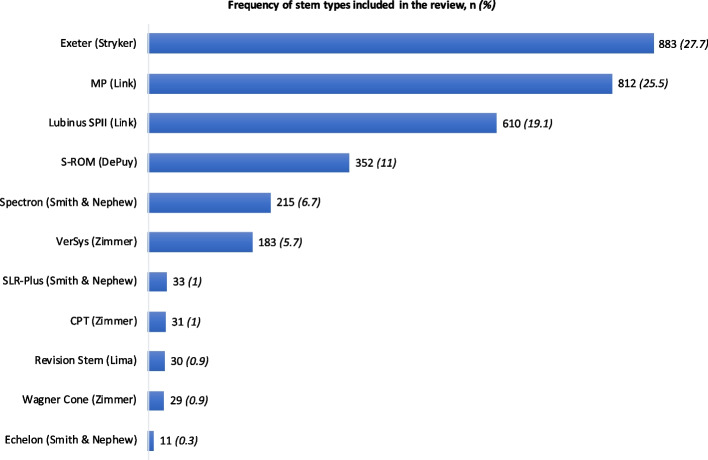
Frequency of femoral stem types and manufacturers encountered in this review

**Fig. 3:**
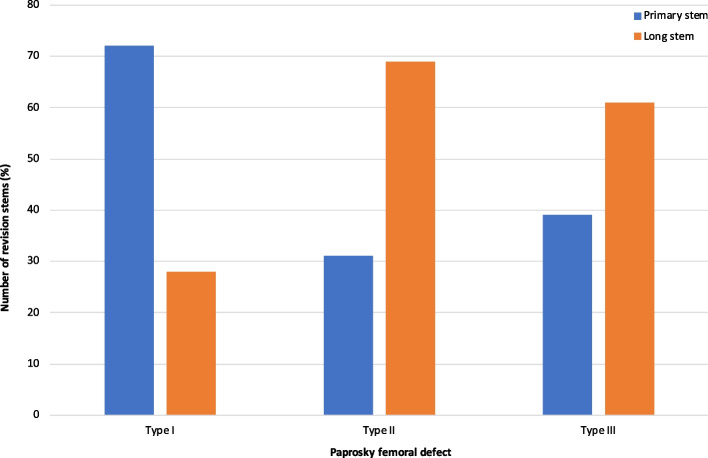
Proportion of primary and long stem usage in Paprosky femoral defect Types I-III

The 9 studies reported on 3,102 rTHAs performed in 2,982 patients with a mean age of 67.4 and a mean follow-up of 5 years (range, 1–15 years). Long stems were used in 1,727 patients (55.7%). rTHA stem fixation across the studies was cementless in 54% and cemented in 46%. For both long and primary length stems, the Exeter (Strkyer; highly polished stainless steel) was most commonly implanted (27.7%), followed by MP (Link, 25.5%; titanium-aluminium-vanadium alloy), Lubinus SPII (Link, 19.1%; cobalt-chrome-molybdenum alloy) and S-ROM (DePuy, 11%; titanium alloy) (Fig. 2). There was a bias for using a primary length stem with Paprosky type I defects (72%), however, with type II and IIIA&B, there was a preference toward using long stems in 69% and 61% of cases, respectively (Fig. 3). Higher reoperation rates were associated extensile femoral osteotomies with primary stems though this was not found to be statistically significant (mean *P* = 0.1). Similarly, there was no significant difference about PJI between groups (mean *P* = 0.6).

All studies stated the indications for rTHA, with aseptic loosening being the most frequent cause for revision (52.7%). Periprosthetic fracture was the second commonest cause for revision (16.1%), followed by PJI (6.3%), recurrent prosthetic dislocation (1.6%) and implant malposition (0.7%). Four studies [21–24] described utilizing an extended trochanteric osteotomy to remove a well-fixed stem or in cases of PJI. Howie et al. [21] also used a transfemoral osteotomy in 13% of their revisions. Four studies did not include femoral osteotomies in their revisions [20, 25, 26, 28]. The study by Weiss et al. [27] was unable to report on osteotomy use due to having data drawn from the Swedish Hip Arthroplasty Register.

Analyzing the stem-related survival, only the studies that reported survival over 5 years were included. We observed a mean value of 89.9% ± 6.7% (SD) for long stems and 91.3% ± 3.5% (SD) for primary length stems. No comparison was performed in the highlighted studies between outcomes from patients with or without osteotomies. Petrie et al. [23] demonstrated encouraging results for patients with PJI treated with primary stem implantation at the second stage in the presence of a healed extended trochanteric osteotomy (ETO) whilst Tetreault et al. [24] reported relatively lower stem survival (90%) with ETO in single stage rTHA.

### Meta-analysis

A total of 6 studies were included in our meta-analysis. The studies included a total of 2,657 patients and 2,681 femora that were operated on.

### Re-revision

**Fig. 4:**
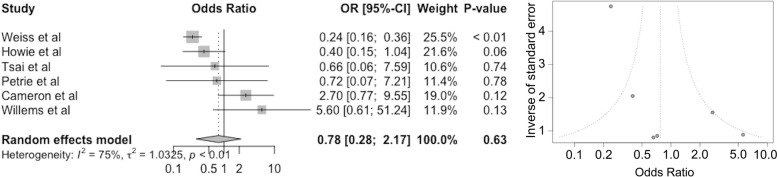
Forest (left) and funnel (right) plots showing the pooled odds ratio (OR, 95% CI) for re-revision in patients after receiving a long stem in revision hip arthroplasty

Of the 6 studies included, 5 reported re-revisions in both long and short-stem groups (Fig. 4). The pooled odds ratio was 0.78 (95% CI 0.28–2.17 *P* = 0.63). The overall re-revision rate was 22% less likely in those in the long stem group. The largest weighted group (Weiss et al. [27]) saw a 76% reduction in re-operations (OR 0.24; 95% CI 0.16–0.36 *P* < 0.01).

### Periprosthetic fractures

**Fig. 5:**
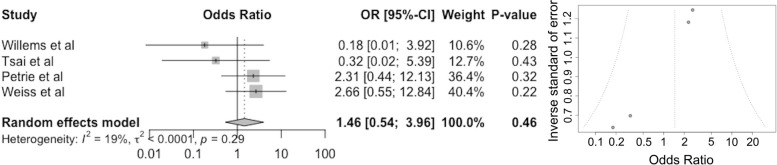
Forest (left) and funnel (right) plots showing the pooled odds ratio (OR, 95% CI) for periprosthetic fractures in patients after receiving a long stem in revision hip arthroplasty

Four studies reported revisions in both long and short-stem groups (Fig. 5). The pooled odds ratio was 1.46 (95% CI 0.54–3.96 *P* = 0.46) (Fig. 5). The overall risk of periprosthetic fractures increased by 46% in the long stem group.

### Dislocations

**Fig. 6:**
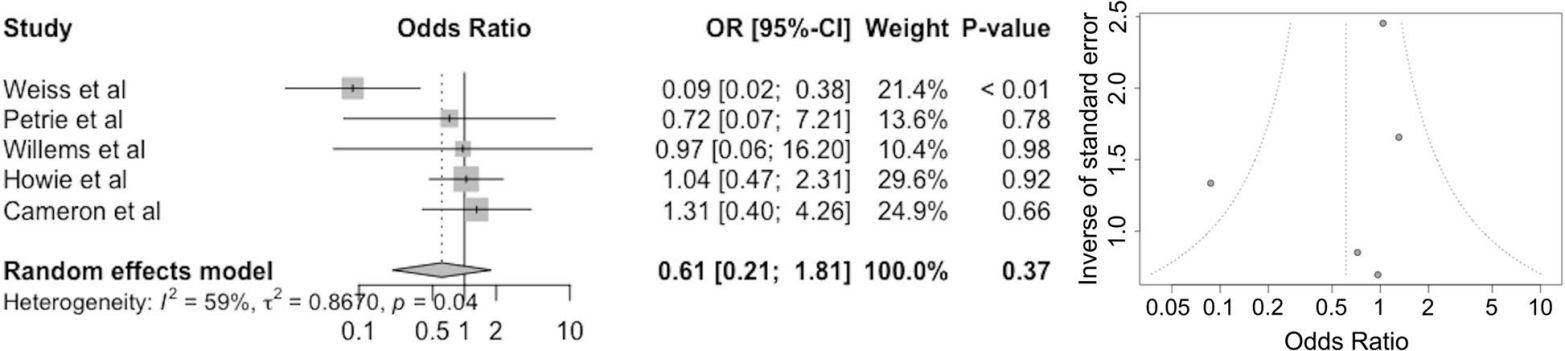
Forest (left) and funnel (right) plots showing the pooled odds ratio (OR, 95% CI) for dislocations in patients after receiving a long stem in revision hip arthroplasty

Five studies reported dislocations in both long and short-stem groups (Fig. 5). The pooled odds ratio was 0.61 (95% CI 0.21–1.81 *P* = 0.37) (Fig. 6). The overall risk of dislocation is 39% lower in the long stem group. The study with the largest weighting (Weiss et al. [27]) showed a reduction of 91% in dislocations in the long stem group (OR 0.09 95% CI 0.02–0.38 *P* < 0.01).

### Functional outcomes

The Harris Hip Score (HHS) was reported in 6 studies and the Oxford Hip Score (OHS) in 1 study. The pooled estimate for HHS in primary length stems was 85.5 (CI 79.8–91.1) and 71.1 (61.2–80.9). The difference of 14.4 points was statistically significant (*P* < 0.05).

## Discussion

Our systematic review demonstrated no significant differences between patient groups about re-operation rates, deep infection, dislocation, pain, and periprosthetic fracture. All studies that reported PROMs favored better HHS for primary stems. However, whilst this difference was statistically significant, it fell below the minimal clinical important difference threshold for HHS [29]. A possible explanation for this may be the younger age and higher levels of activity of these patients receiving shorter revision stems.

On the basis of laboratory and biomechanical tests, Retpen and Jensen [30] reported improved fixation in vivo if the revision stem over-bridged the previous stem by 1 diaphyseal width, suggesting that cementing into a newly opened cancellous bed was critical for implant stability. Berry et al. [31] stated that the revision stem should extend at least 2 cortical diameters beyond the tip of the previous implant, which has been an accepted rule of thumb when sizing an appropriate length of revision stem. More recently, however, having adequate fixation “as proximal as possible and as distal as necessary” is gaining attention in an effort to reduce the proximal bone resorption that can arise from distal fixation alone [13, 32, 33]. Established literature suggests that in the presence of an ETO, the revision femoral component must be long and bypass the prior stem. However, there is a paucity of studies regarding the survivorship of different osteotomies [34, 35]. Tetreault et al. [24] reported a lower implant survival rate (90%) with ETO and further work by Cavagnaro et al. [16] concluded that ETO should be considered a relative contraindication for using primary length stems. Conversely, there is a considerable difference between ETO or transfemoral osteotomy and a limited cortical window. We would suggest using a primary stem for the latter.

In brief, the decision-making on which revision stem to use not only comes down to the expertise, familiarity, and preference of the surgeon but is significantly influenced by the ability of the femur to support a stable implant. Whilst many elements, such as stem material and geometry, influence prosthesis survivorship and patient outcomes, the choice of stem, modularity, and fixation method are critical factors when planning revision arthroplasty [34]. With regard to modularity, a systematic review by Koutalos et al. [36] found no differences between monoblock and modular stems in terms of reoperation, dislocation, periprosthetic fracture or infection rates though monoblock stems exhibited more subsidence whilst modular stems displayed higher stress shielding and intraoperative fracture rates in accordance with our results. Whilst modular stems were introduced to better restore length and offset without compromising axial stability, there is a recognized association with intraoperative fractures compared to monoblock options. A greater variation in stem axis geometry and taper angle may explain this event. Huang and Huddlestone reported an 11% rate of periprosthetic fractures in Paprosky I-IIIA revisions with a modular revision stem [10, 37]. Furthermore, a higher fracture risk may be explained by the fact that these stems are often used in femora with greater bone loss and thus are more susceptible to fracture. More recently, modularity has been linked with trunnionosis and taper corrosion which can cause junctional failure as well as adverse local reaction to metallic debris [38, 39]. Therefore, given similar survivorship and functional outcomes, monoblock stems may be a more appealing option for non-complex revision.

Concerning stem fixation, registry data suggest that uncemented femoral stems have a higher early all-cause revision rate than cemented stems in primary arthroplasty, with similar trends in rTHA [40–43]. Despite this, the use of uncemented revision stems continues to rise in these registers. In line with our findings, register studies also indicated that cemented revision stems may fare better in older patients with poor bone stock and with shorter life expectancy. However, these register studies lack information on the size of the femoral defect, femoral anatomy and general health of the patient, which can significantly influence surgical decision-making [44]. Patient age also plays a key factor and given that younger patients undergoing first revision with an uncemented stem will have a significant lifetime risk of all-cause failure, older patients undergoing cemented rTHA may contribute to more favorable results for using cement. Abdel et al. [45] identified a three-fold increased risk of intraoperative periprosthetic fracture with uncemented stems (19%) compared with cemented stems (6%) in rTHA. In addition, Hernigou et al. [46] recommend the use of long cemented stems in older patients with severe bone loss and previous revision, with significantly fewer periprosthetic fractures and early revisions when compared with primary uncemented stems.

In the case of revision for periprosthetic fracture, particularly in the presence of a non-supportive metaphysis, it is common to implant a longer, diaphyseal-engaging stem. Nonetheless, if anatomical reconstruction is achievable, internal fixation of periprosthetic fracture around a femoral component is associated with significantly lower reoperation rates and lower critical care and transfusion requirements compared to revision arthroplasty [47]. Fixation or revision arthroplasty for a periprosthetic fracture is equally challenging and, often, the decision-making depends on surgeon expertise, implant availability and patient factors. Although the studies in this review do not expand and correlate these variables to their outcomes, Toci et al. [48] reported that surgeon experience exerts a significant influence on reoperation rates in revision for Vancouver B fractures.

This review included 9 studies conducted across separate institutions and involved patients from a wide demographic range. Therefore, the results are likely to be generalizable to the wider population. Nonetheless, there are a few limitations that require acknowledgment. The majority of studies were of level II evidence and only 1 RCT was included in this review. Secondly, we judged a high-to-moderate degree of heterogeneity between studies in the meta-analysis, which likely reflects the nature of the studies that tend to report on outcomes from a single centre. Heterogeneity extends further to the variety of implants included in this review. The range of metal alloys and stem coatings differ in their moduli of elasticity and also stress shielding to local bone. Furthermore, our review included a mixture of full and femur-only revisions. Whilst it would be better to compare femur-only revisions, this has not been feasible based on the limited literature. Few studies reported on the patient’s pre-morbid status, such as the American Society of Anesthesiologists (ASA) grade, which may influence surgical planning and overall survivorship. In addition, differences in known risk factors for surgical site infection between patient groups could not be excluded. Finally, our review only included articles in the English language and may not represent all the published literature on this subject.

## Conclusion

On the basis of moderate quality evidence, we recommend that a primary length stem affords non-inferior outcomes in rTHA when compared with longer stems with regards to risks of major complications, such as further re-operation, dislocation, infection and functional outcomes. A primary stem preserves distal bone stock which may be of benefit to younger patients whilst also being a more cost-effective and potentially straightforward procedure. If there are concerns regarding a non-supportive metaphysis then diaphyseal engagement with a long-stemmed implant should be considered. In older patients who would benefit from a decreased risk of complications and with lower functional demands, we recommend using a long cemented stem. The justifications for using monoblock or modular implants as well as the method of fixation should be considered when counselling patients.

## Data Availability

Data sharing is not applicable to this article as no datasets were generated or analyzed during the current study.

## Abbreviations

THA: Total Hip Arthroplasty
rTHA: Revision Total Hip Arthroplasty
PROMs: Patient Reported Outcome Measures
HHS: Harris Hip Score
PJI: Prosthetic Joint Infection
OR: Odds Ratio
CI: Confidence Interval
AL: Aseptic Loosening
GT: Greater Trochanter
LLD: Leg Length Discrepancy
PC: Prospective Cohort
RC: Retrospective Cohort
RD: Recurrent Dislocation
PPFx: Periprosthetic Fracture
ETO: Extended Trochanteric Osteotomy
OHS: Oxford Hip Score
RCT: Randomised Controlled Trial
ASA: American Society of Anaesthesiologists
